# Biological Control of Fungal Diseases by *Trichoderma aggressivum* f. *europaeum* and Its Compatibility with Fungicides

**DOI:** 10.3390/jof7080598

**Published:** 2021-07-24

**Authors:** Brenda Sánchez-Montesinos, Mila Santos, Alejandro Moreno-Gavíra, Teresa Marín-Rodulfo, Francisco J. Gea, Fernando Diánez

**Affiliations:** 1 Departamento de Agronomía, Escuela Superior de Ingeniería, Universidad de Almería, 04120 Almería, Spain; brensam@hotmail.com (B.S.-M.); alejanmoga@gmail.com (A.M.-G.); tere_rodu@hotmail.com (T.M.-R.); 2 Centro de Investigación, Experimentación y Servicios del Champiñón (CIES), Quintanar del Rey, 16220 Cuenca, Spain; fjgea.cies@dipucuenca.es

**Keywords:** biological control, *Trichoderma*, fungal phytopathogens, diseases, fungicides

## Abstract

Our purpose was to evaluate the ability of *Trichoderma aggressivum* f. *europaeum* as a biological control agent against diseases from fungal phytopathogens. Twelve isolates of *T. aggressivum* f. *europaeum* were obtained from several substrates used for *Agaricus bisporus* cultivation from farms in Castilla-La Mancha (Spain). Growth rates of the 12 isolates were determined, and their antagonistic activity was analysed in vitro against *Botrytis cinerea*, *Sclerotinia sclerotiorum*, *Fusarium solani* f. *cucurbitae*, *Pythium aphanidermatum*, *Rhizoctonia solani,* and *Mycosphaerella melonis*, and all isolates had high growth rates. *T. aggressivum* f. *europaeum* showed high antagonistic activity for different phytopathogens, greater than 80%, except for *P. aphanidermatum* at approximately 65%. The most effective isolate, *T. aggressivum* f. *europaeum* TAET1, inhibited *B. cinerea*, *S. sclerotiorum,* and *M. melonis* growth by 100% in detached leaves assay and inhibited germination of *S. sclerotiorum* sclerotia. Disease incidence and severity in plant assays for pathosystems ranged from 22% for *F. solani* to 80% for *M. melonis*. This isolate reduced the incidence of *Podosphaera xanthii* in zucchini leaves by 66.78%. The high compatibility by this isolate with fungicides could allow its use in combination with different pest management strategies. Based on the results, *T. aggressivum* f. *europaeum* TAET1 should be considered for studies in commercial greenhouses as a biological control agent.

## 1. Introduction

In the past few decades, the use of plant growth-promoting microbes (PGPMs) and microbial biological control agents (MBCAs) has greatly increased in agriculture, mainly because consumers and producers are interested in reducing synthetic organic pesticides and chemical fertiliser residues in food, and in reducing the environmental impact of agricultural production. The extensive use of synthetic pesticides since 1945 has resulted in long-term environmental problems [1] and high health risks for humans [2]. About 3 to 4.6 million tons of pesticides are used annually, and the global intensive use of chemical fertilisers was about 109.1, 45.5, and 37.6 million tons of N, P_2_O_5_, and K_2_O, respectively, in 2017 (35.9% average increase since 2002) [3,4,5]; it directly endangers soil and water resources, causes environmental pollution [6] and inflicts diseases at alarming levels [7]. Therefore, sustainable agriculture will be achieved by biological pest and fertiliser management strategies; both are more natural and environmentally friendly alternative solutions to manage crop production with reduced use of pesticides and fertilisers.

Among the numerous microbes used as PGPMs or MBCAs (also termed probiotics), bacterial or fungal strains are the most frequently used against causal agents of aerial and soil diseases in plants. However, *Trichoderma* remains the gold standard of biological control. Numerous species of this genus, including *T. harzianum* Rifai; *T. viride* Pers; *T. atroviride* P. Karst; *T. virens* J.H. Mill, Giddens and A.A. Foster; *T. longibrachiatum* Rifai; *T. polysporum* Rifai; *T. stromaticum* Samuels and Pardo-Schulth; *T. hamatum* Bainier; *T. asperellum* Samuels, Lieckf and Nirenberg; *T. citrinoviride* Bissett; *T. saturnisporum* Hammill; *T. aggressivum* Samuels and W. Gams have been described as biological control agents (BCAs) against phytopathogens [8,9,10,11,12,13,14,15,16,17,18,19,20,21,22,23,24]. Several studies have shown that species in this genus present most mechanisms identified in biological control, namely competition for resources (space and nutrients) [25], production and excretion of metabolites (antibiotics, cell wall-degrading enzymes, siderophores) [26,27,28,29,30], mycoparasitism on phytopathogenic fungi [31], and induction of defence responses (systemic acquired resistance, induced systemic resistance, and hypersensitive response) [32,33,34,35,36,37]. Among these, competition, mycoparasitism, and the production of antifungal compounds are considered the most important mechanisms [38,39]. Mycoparasitism is based on the recognition, binding, and rupture of the cell wall of the host fungus by numerous enzymes, primarily chitinolytic and glucanolytic enzymes [28,40]. As a result of mycoparasitism, many species of *Trichoderma*, including those indicated above, have been identified as causative agents of the disease known as green mould in mushroom crops in different countries [41,42,43,44,45,46,47]. *T. pleurotum* and *T. pleuroticola* have been detected in *P. ostreatus* [48], *T. aggressivum* in *Agaricus bisporus* [49], and *T. harzianum, T. longibrachiatum, Trichoderma ghanense, T. asperellum*, and *T. atroviride* in *Agaricus* compost substrates and *Pleurotus*, although they are not particularly aggressive [41]. One of these species, *T. aggressivum* f. *europaeum* Samuels & W. Gams, previously known as *T. harzianum* Th2 biotype, is responsible for *Agaricus* green mould problems in Europe [50]. Hyphal coiling and the formation of appressoria like structures is usually associated with mycoparasitism by *Trichoderma* species [32]. However, this kind of interaction between *T. aggressivum* and *A. bisporus* has rarely been observed [51]. Its most successful mechanism of action is its competitive tolerance to the inhibitory effect of numerous bacteria and fungi, which accounts for its rapid growth and sporulation [52,53], although *T. aggressivum* can be considered a mycopathogen given its effect on *A. bisporus* hyphae, as well as its induction of an oxidative stress response [54]. O’Brien et al. [55] reported a change in the production of intracellular proteins in the presence of *A. bisporus* directly related to stress tolerance, cell signalling, longevity, and structure. These functions may be part of the capability of *T. aggressivum* to displace *A. bisporus* and to decrease mushroom performance. It grows quickly (1 mm h^−1^) at 27 °C, producing a cottony layer of aerial mycelium. Sporulation starts only after 4 d and occurs in the central area, in concentric green bands. This species efficiently competes for space and nutrients, and produces extracellular enzymes, toxic secondary metabolites, and volatile organic compounds, resulting in drastic crop losses.

As for other *Trichoderma*, this species can be considered a biological control agent or a plant growth promoter. Accordingly, Ordaz-Ochoa et al. [56] described mycelial growth inhibition in dual cultures of different *Armillaria* spp. isolates from avocado by *T. aggressivum*. Recently, Sánchez-Montesinos et al. [24] highlighted the ability of *T. aggressivum* f. *europaeum* to promote melon plant growth under saline conditions. These authors also reported the damping-off control caused by *Pythium ultimum* in melon seedlings after applying *T. aggressivum* f. *europaeum,* reducing disease severity by 63%. Rodriguez et al. [57] have isolated and identified *T. aggressivum* as mycoparasites on coffee rusts caused by *Hemileia vastatrix*, which was then described for the first time in the tropics. This benefit has also been observed in the germination and development of tomato and pepper seedlings, leading to increased root dry weight by 66.66% and 36.36%, respectively [58]. Similarly, Lee et al. [59] have reported *Arabidopsis* growth promotion mediated by volatile compounds produced by *T. aggressivum*, with an increase of 37.1% in fresh shoot weight and 82.5% in chlorophyll. However, little is known about the role that *T. aggressivum* may play in fighting different phytopathogens both in vitro and in vivo.

Therefore, the main goals of this study are to (a) isolate and select *T. aggressivum* f. *europaeum* (TAE) strains, obtained from substrates of *A. bisporus* cultures and from carpophores with green mould, (b) determine the in vitro antagonistic activity of TAE isolates against different phytopathogens of interest in agriculture, (c) assess the in vivo capacity for controlling the development of the disease caused by phytopathogens in different pathosystems under greenhouse conditions, and (d) determine the compatibility of TAE with different fungicides commonly used to control fungal diseases.

## 2. Materials and Methods

### 2.1. Isolation and Identification of Trichoderma Isolates

**Figure 1:**
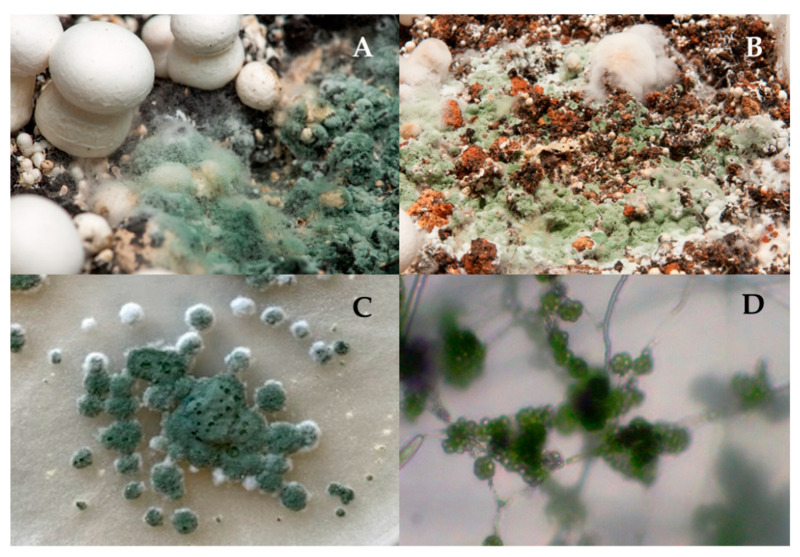
Green mould on the casing layer and mushroom (**A**,**B**). Isolate of *T. aggressivum* f. *europaeum* TAET1: Culture (**C**) and conidiophores (**D**).

A total of 12 isolates were obtained from mushroom farms located in Castilla-La Mancha (Spain) showing green mould disease symptoms (Figure 1). The isolates were maintained on potato dextrose agar (PDA) medium at 25 °C in the dark and were characterised taxonomically and molecularly. Conidiophore morphology was examined by light microscopy and were consistent with the genus *Trichoderma* (Figure 1).

Molecular identification was performed using the procedure of Carrasco et al. [60]. Sequencing of the rDNA region, including the spacers ITS1 and ITS2 and 5.8S rDNA, was conducted by automated DNA sequencing with fluorescent terminators using an ABI 377 Prism Sequencer (Applied Biosystems, Foster City, CA, USA) by the Technical Service of University of Almería. ChromasPro^®^ (Technelysium Pty. Ltd., Tewantin, Australia) analysis software was used to edit each sequence sample. The obtained sequences were compared with those registered in the GenBank (NCBI) database through MegaBLAST searches for identification [61,62]. Sequences were deposited in the EMBL database.

### 2.2. Growth of the Trichoderma Isolates in PDA

The growth of each isolate was evaluated in vitro by placing a 5 mm plug from the edge of a 7 d old pure PDA culture, 0.5 cm from the edge of the Petri dish. The isolates were incubated in a growth chamber at 25 °C in the dark. The colony diameters were recorded every day during culturing. The experiment was completely randomised with five replicates.

### 2.3. Dual Culture Test

*Trichoderma* isolates were screened for their antagonism against *B. cinerea* Pers, *S. sclerotiorum* (Lib.) de Bary, *F. solani* f. *cucurbitae* W.C. Snyder & H.N. Hansen, *P. aphanidermatum* (Edson) Fitzp, *R. solani* J.G. Kühn and *M. melonis* (Pass.) by adopting the confrontation assay of Santos et al. [63]. Petri dishes (9 cm diameter) containing 17 mL of PDA (Bioxon, Becton Dickinson, Mexico) were prepared. Then, 0.5-cm plugs of mycelium of all fungi were cut from the growing edge of seven days old cultures with active growth of each isolate. The plugs were placed at the ends of Petri dishes with a distance of 7.5 cm between the two fungi, antagonist–phytopathogen. All plates were sealed with Parafilm^®^ and incubated in the dark at 25 °C during 3 to 10 days. Radial fungal colony growth was measured daily. Results were transformed into percentages of mycelium growth inhibition (PIRM: percentage inhibition of radial mycelia growth of the pathogen, R1: radial growth of pathogen in control plates, R2: radial growth of pathogen in dual culture plates). These tests were conducted in quintuplicate. Zones where antagonist and pathogen meet were also observed by light microscopy and cryo-fracturing electron scanning microscopy (cryo-sem) according to the procedure of Diánez et al. [23]. Only one isolate was selected for subsequent tests, that is, the isolate with the highest growth rate, as well as a greater antagonistic capacity against a greater number of phytopathogens.

### 2.4. Antifungal Volatile Organic Compounds Bioassay

In vitro inhibition effects of volatile organic compounds produced by *T. aggressivum* f. *europaeum* TAET1 against different phytopathogenic fungi was determined. For this, a disk (0.5 cm diameter) was cut from the actively growing edges of TAET1 plates and was placed at a distance of 0.5 cm from the edge onto one half of a divided plate containing PDA medium. Following a 48 h incubation period at 25 ± 1 °C, 0.5 cm mycelial plugs for each pathogen were placed on the other half of the divided plate, containing PDA, and the plates were immediately wrapped in Parafilm^®^. Measurements of radial mycelial growth were taken daily for fungi with rapid growth (*B. cinerea*, *S. sclerotiorum*, *P. aphanidermatum,* and *R. solani*) and every 2 d for those growing more slowly (*M. melonis* and *F. solani*) until the edge of the plate was reached [23]. The experiment was performed five times with five replicates.

### 2.5. Detached Leaf Assay

Suppressive effects of *T. aggressivum f. europaeum* TAET1 on *B. cinerea, S. sclerotiorum* and *M. melonis* were assessed using a detached leaf assay as described by Novak et al. [64] and Patial et al. [65]. Leaves of cucumber (variety, Super Marketer, Mascarell), pepper (var. Pimiento del Padrón, Mascarell) and tomato (var. Red Cherry, Fitó) seedlings were disinfected using 3% sodium hypochlorite for 30 s and were washed twice to remove residues. The leaves were immersed for 3 min in a solution containing TAET1 spores at a dose of 10^6^ spores·mL^−1^ or were immersed in an aqueous solution containing the fungicide Switch (cyprodinil 37.5% and fludioxonil 25% (WG) *w*/*w*; Syngenta, Basel, Switzerland) at 600 ppm to compare efficacy. Whole leaves or fragments were then placed on wet filter paper in plastic trays, and the centre of the leaf or leaf fragment was carefully punctured using a sterilised needle. A 0.5-cm disk containing the corresponding pathogen was placed at the puncture site. Petri dishes were then incubated at 25 °C, and number of leaves with symptoms were counted and photographed 72 h after inoculation. This experiment was repeated twice.

### 2.6. Degradation of Sclerotia of Sclerotinia Sclerotiorum by T. aggressivum f. europaeum

TAET1 were evaluated for efficacy in colonising and destroying sclerotia in soil. Soil moistened to field capacity was sterilised at 120 °C for 60 min twice on two consecutive days. Sclerotia were disinfected with 1% NaOCl for 2 min. Five sclerotia (0.5–1 cm) were mixed with the soil and then transferred to a Petri dish. Cultures of TAET1 were grown on PDA for 7 d. Soil was infested with a 0.5 cm plug placed on the soil surface. The plates were kept at 25 °C in the dark. After 25 d, the sclerotia were recovered from the soil and rinsed in tap water. The number of recovered sclerotia and the presence of white mycelium on its surface were determined. The experiment was performed twice with five replicates.

### 2.7. Compatibility of T. aggressivum f. europaeum TAET1 with Fungicides

In vitro compatibility of TAET1 with different selected fungicides (Table 1) for mycelial growth inhibition was established using the poisoned food technique [66,67]. The minimum recommended dose (D2), the maximum recommended dose (D3), 0.5 × D2, and 1.5 × D3 (Table 1) of each fungicide were tested. Using a sterile cork borer, mycelial discs (0.5 cm diameter) were cut from actively growing seven-days-old fungal cultures and were placed in the centre of a Petri dish containing PDA supplemented with various pesticides. Five replicates were used per treatment. Fungicidal or fungistatic effects of each fungicide was determined by transferring the initial disk of TAET1, which did not grow with fungicide to a PDA plate without fungicide.

Toxicity, i.e., compatibility of TAET1 and the fungicide, was classified using the scale of the International Organisation for Biological Control (OILB) [68]. This classification groups compatibility between microorganisms and fungicides depending on the proportion of inhibition compared to a control (<30%: harmless; 30–75%: slightly toxic; 75–90%: moderately toxic; >90%: toxic).

### 2.8. Evaluation of T. aggressivum f. europaeum on the Severity of Seven Phytopathogens

Biocontrol effects of TAET1 on different pathosystems was determined: *B. cinerea*–melon, *S. sclerotiorum*–pepper, *R. solani*–tomato, *F. solani*–zucchini, *P. aphanidermatum*–melon, *M. melonis*–melon, and *P. xanthii*–zucchini.

Seeds were disinfected using 2% hypochlorite for 3 min and were washed thoroughly with tap water to eliminate residues. Subsequently, the seeds were sown in 500 mL pots containing a commercial peat mix, at one seed per pot.

Then, 5 mL spore suspension of TAET1 was added to each pot at 1 × 10^6^ propagules/plant; the control treatments received 5 mL water. To assess diseases of aerial parts, foliar spraying was carried out at the same dose to wet the whole plant with the TAET1 solution. Spraying was carried out three days before applying conidia/mycelium of the respective pathogen.

To prepare *P. aphanidermatum* inocula, the procedure described by Marin et al. [69] was followed. Inocula of the other phytopathogens were prepared by scraping and subsequent filtration, apart from *R. solani*, *S. sclerotiorum*, and *P. xanthii*. Phytopathogens were inoculated when the plants showed a second true leaf, and using a sterile micropipette, inoculation was performed by uniformly applying the zoospores/conidia suspension (5 mL) at a concentration of 10^4^ CFU·mL^−1^ uniformly to the peat surface. In the case of *B. cinerea* and *M. melonis*, the pathogen was applied by spraying the plant five times at the same concentration. Before this, the first true leaf had been cut to facilitate pathogen entry.

Inoculation with *R. solani* was carried out by mixing mycelium into the substrate; inoculation with *S. sclerotiorum* was performed using the spray mycelium method as described by Chen and Wang [70]. The stem was wounded to facilitate pathogen entry. Symptom severity was recorded continuously and 30–60 days after inoculation, a final disease severity index was estimated according to the following scale: 0 = healthy plant; 1 = initial symptoms; 2 = moderate symptoms (25%); 3 = affected plant (50%); 4 = severely affected plant (75%); and 5 = dead plant.

In addition, a second trial to test the biocontrol effect of TAET1 on the pathosystem *Fusarium solani* f. *cucurbitae*-zucchini, using two different doses 10^5^ (F1) and 10^4^ (F2) CFU·mL^−1^, was carried out.

To determine the control effect of TAET1 on powdery mildew, an inoculum of *P. xanthii* was prepared from field-collected zucchini leaves affected by cucurbit powdery mildew. Using a sprayer, sterile distilled water was sprayed under pressure to rinse of fungal conidia. The suspension was collected, and experimental plants were immediately inoculated at a concentration of 10^4^ CFU·mL^−1^. To determine the suppressive effect of TAET1 on the disease, the leaf area affected by powdery mildew was determined using WinDIAS 3.1.lnk software (Dynamax, Fresno, CA, USA) to calculate the proportion of affected leaf area with respect to total leaf area. Additionally, the numbers of affected leaves and petioles per plant were counted.

All pathogenicity tests were performed under greenhouse conditions and at different seasons to provide optimal environmental conditions for each pathogen. Experimental units consisted of four repetitions with 24 plants per pathosystem. Two experiments were conducted using a completely randomized block design.

### 2.9. Statistical Analyses

Data were analysed using analysis of variance with Statgraphics Centurion version XVI software. Mean separation was carried out using Fisher’s least significant difference test. Data were tested using a one-way analysis of variance or Student’s *t*-test; statistical significance is reported at *p* < 0.05.

## 3. Results

### 3.1. Morphological and Molecular Identification

The macro and microscopic observations of all isolates from mushroom farms confirmed that the different isolates belonged to the genus *Trichoderma* (Figure 1). The results of the identity analysis of the sequences obtained for the 12 strains allowed confirmation that they belonged to *T. aggressivum* f. *europaeum*.

### 3.2. Growth of Trichoderma Isolates

**Figure 2:**
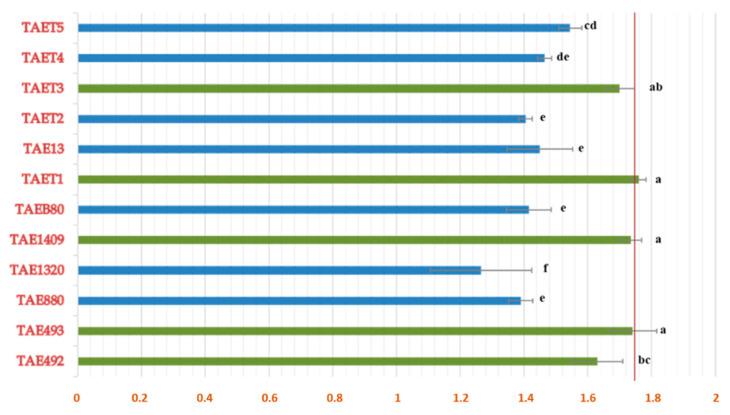
Growth rate (cm d^−1^) of different *T. aggressivum* f. *europaeum* isolates. Different letters indicate significant differences according to the one-way ANOVA (*p* = 0.05). (Accession number TAET1: MW751677.1).

In Figure 2, the results obtained from the mycelial growth of 12 isolates of *Trichoderma aggressivum* f. *europaeum* are shown. The colony growth of most isolates was fast, reaching the opposite end of the Petri dish within 96–120 h. The isolates TAET1, TAE493, and TAE1409 were the fastest, with a growth rate of 1.76, 1.74 and 1.73 cm d^−1^, respectively. No isolates were discarded in determining the antagonistic activity of *T. aggressivum* against phytopathogens.

### 3.3. Effects of T. aggressivum f. europaeum Isolates on the Radial Growth of Phytopathogens

The results from the comparison of the 12 *T. aggressivum* f. *europaeum* (TAE) isolates to the different phytopathogens are shown in Table 2.

**Figure 3:**
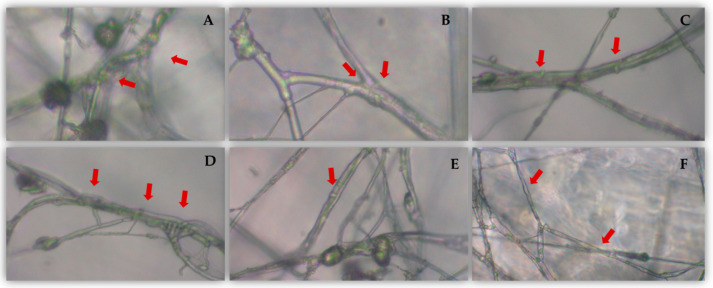
Mycoparasitism by *Trichoderma* hyphae a coiling around the hyphae (**A**–**E**). Hook formation (**C**,**E**), wrapping (**B**,**D**) and hyphallysis (**F**). Images were taken with a × 40 objective.

All TAE isolates showed high in vitro antagonistic activity against all phytopathogenic fungi tested. The highest inhibition percentages were detected for *F. solani* and *M. melonis*, which reached values close to 90%. In contrast, the lowest inhibition values were detected for *P. aphanidermatum*, with inhibition ranging from 53 to 65%. For the other phytopathogens, mycelial growth inhibition ranged from 70 to 90%. *Trichoderma* mycoparasitism process with hyphal coiling around the phytopathogens was observed (Figure 3).

One isolate was selected for the subsequent tests, TAET1, because it showed the highest growth rate and inhibition percentage for some phytopathogens.

### 3.4. Effect of Volatile Compounds on the Mycelial Growth of Phytopathogenic Fungi

The effect of volatile metabolites on the growth of phytopathogens is shown in Table 3. No significant inhibition percentages were found for most fungi, except for *F. solani* and *M. melonis*, which showed a decrease in growth in the last days, with mycelial inhibition percentages of 12.85% and 18.60%, respectively.

### 3.5. Detached Leaves Assay

**Figure 4:**
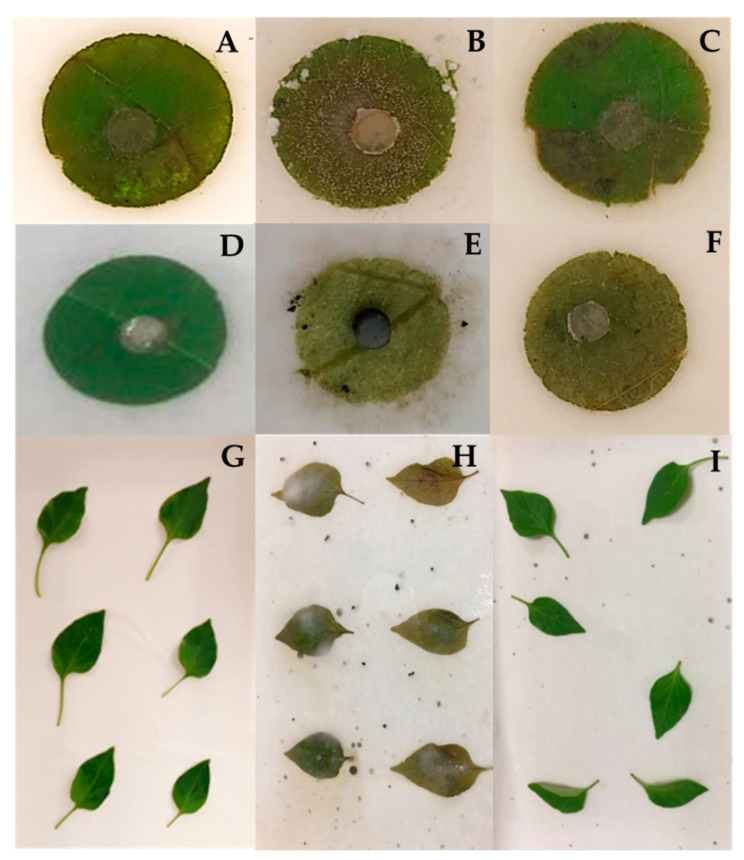
Symptoms of *B. cinerea* (**A**–**C**), *M. melonis* (**D**–**F**) and *S. clerotiorum* (**G**–**I**), infection on detached leaves treated with TAET1 (**A**,**D**,**G**), fungicide (**C**,**F**,**I**) or control (**B**,**E**,**H**).

Applying TAET1 spores on cucumber, pepper, and tomato leaves prevented the onset of symptoms caused by *B. cinerea*, *S. sclerotiorum,* and *M. melonis* in 100, 100, and 93% of the leaves, respectively, with the same efficacy as the control fungicide. Leaves not inoculated with TAET1 showed mycelial growth and obvious symptoms of rotting in 100% of the leaves for the three phytopathogens tested in this study (Figure 4).

### 3.6. S. sclerotiorum Sclerotia Degradation

**Figure 5:**
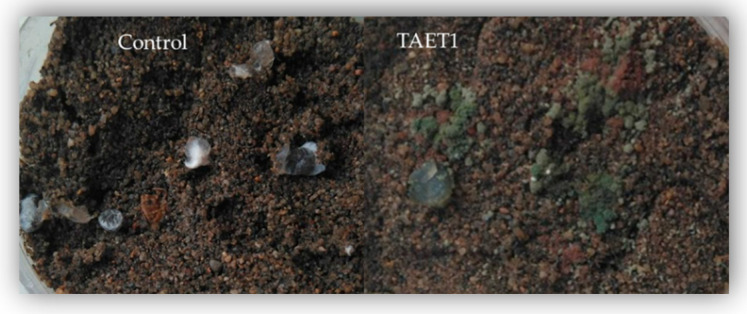
Effect of TAET1 on *sclerotial germination.* Control: sclerotia with white mycelium on their surface. TAET1: Observation of *Trichoderma on soil surface* (green mass).

Applying *T. aggressivum* f. *europaeum* TAET1 resulted in a marked decrease in sclerotia viability (Table 4). Sclerotia treated with TAET1 did not show white mycelium on their surface or TAET1 conidiophores. Similarly, TAET1 sporulation was detected on the soil surface (Figure 5).

### 3.7. T. aggressivum f. europaeum Compatibility with Fungicides

The observed effects of 19 fungicides on growth and development of *T. aggressivum* f. *europaeum* TAET1 are presented in Table 1. The results indicated that mycelial growth of TAET1 was affected by the different doses of each of the fungicides tested in vitro, compared to the control. According to the OILB scale [68], the compatibility of the 19 fungicides tested using minimum (D2) and maximum (D3) recommended doses in horticultural crops was as follows: three were harmless (kresoxim-methyl, pencycuron and cymoxanil; inhibition <30%), four were slightly toxic (flutriafol, azoxystrobin, fenhexamid, fosetyl-Al; 30–75%), one was moderately toxic (folpet + metalaxyl-M; 75–90%), and four were toxic (thiophanate-methyl, pyrimethanil, mancozeb and chlorothalonil; >90%). Total growth inhibition at doses D2 and D3 were observed only in two fungicides (thiophanate-methyl and mancozeb) with fungicidal effects.

Seven fungicides showed different behaviour according to the scale at maximum and minimum doses; four (iprodione, triadimenol, propamocarb, and dimethomorph + mancozeb) were slightly toxic at the maximum doses (D3) and harmless at the minimum doses (D2); two (myclobutanil and copper oxychloride) were moderately toxic at (D3) and slightly toxic at (D2); and one (cyprodinil + fludioxonil) was toxic at (D3) and moderately toxic at (D2).

### 3.8. Biocontrol of T. aggressivum f. europaeum against Fungal Diseases

**Figure 6:**
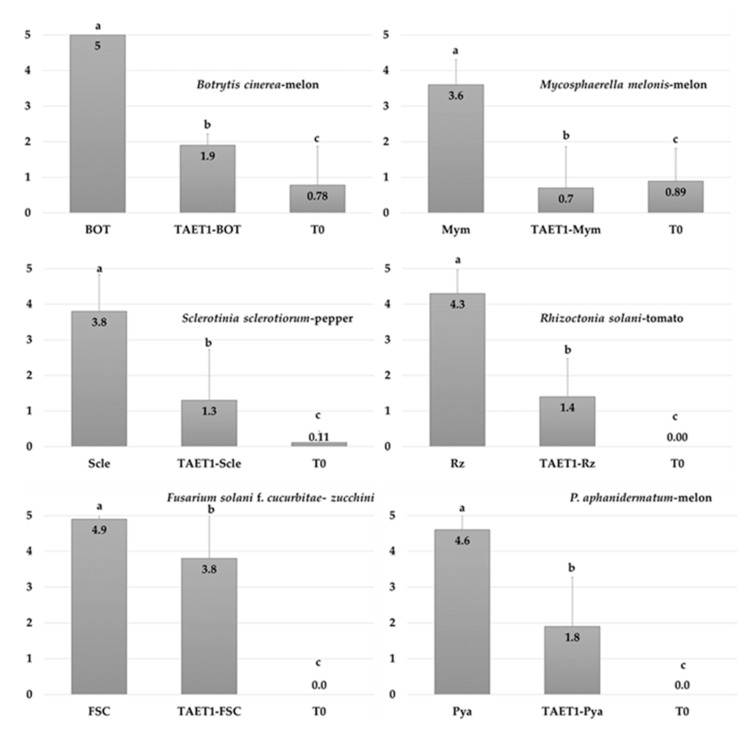
Disease severity of phytopathogens in plants was rated 30–90 d after inoculation based on a 0–5 scale: where 0 = no visible disease symptoms and 5 = plant dead. T0: control; TAET1: *T. aggressivum f. europaeum* TAET1; Bot: *B. cinerea*; Mym: *M. melonis*; Scle: *S. sclerotiorum*; Rz: *R. solani*; FSC: *F. solani* f. *cucurbitae*; Pya: *P. aphanidermatum*. Mean standard deviation is expressed by error bars (24 plants per repetition). Means with the same letter are not significantly different (LSD) according to the ANOVA (*p* < 0.05).

*T. aggressivum* f. *europaeum* TAET1 decreased the disease severity in all study pathosystems (Figure 6).

**Figure 7:**
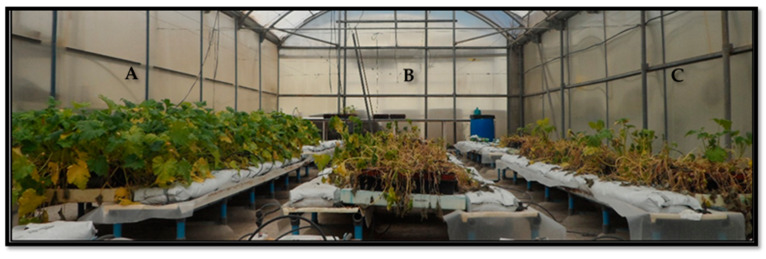
Comparison of the state of the plants at the end of the test: without FSC inoculum (**A**) and the treatments with F1 (**B**) and with F2 (**C**), with TAET1. FSC: Control without *F. solani* f. *cucurbitae* (FSC). F1: 10^5^ CFU·mL^−1^; (F2): 10^4^ CFU·mL^−1^.

**Figure 8:**
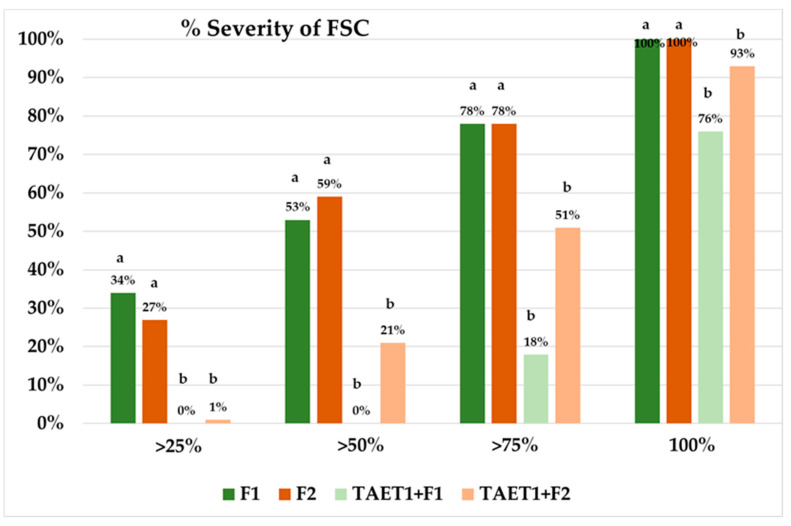
Comparison of the TAET1 effect on the percentage of disease severity after applying two doses of FSC (F1: 10^5^ CFU mL^−1^; F2: 10^4^ CFU mL^−1^. 25%, 50%, 75%, and 100%: disease severity percentages higher than 25%, 50%, 75%, and 100% for F1 and F2. Means with the same letter are not significantly different (LSD) according to the ANOVA (*p* < 0.05).

The strongest effect was observed on *M. melonis*, with an 80.55% decrease in disease incidence. In *B. cinerea* and *S. sclerotiorum*, the decrease in disease severity reached 62% and 65.78%. Disease was also observed in the uninoculated controls (T0) of some of the pathosystems, because of the random arrangement of treatments. The severity of diseases caused by *P. aphanidermatum*, *R. solani,* and *F. solani* f. *cucurbitae* decreased by 58.69%, 67.44%, and 22.44%, respectively, after applying TAET1 spores (Figure 6). Two weeks after the last evaluation, no changes were observed in the severity index of the different pathosystems, except for *F. solani* f. *cucurbitae*, in which all plants reached mortality index 5. Repeating the test for *F. solani* f. *cucurbitae* with two inoculum concentrations confirmed the delay in disease onset, after applying TAET1, albeit without controlling the disease (Figure 7). Despite the high inoculum pressure, when the disease incidence was higher than 75%, applying TAET1 reduced the disease severity by 77 and 34%, upon F1 and F2 inoculum doses, respectively (Figure 8).

**Figure 9:**
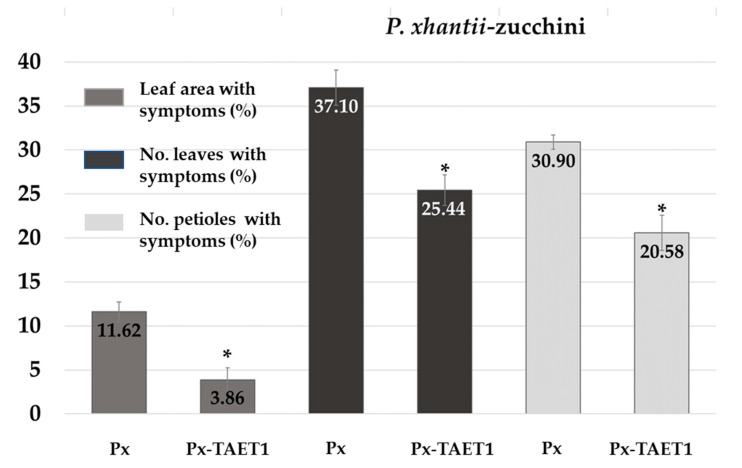
Control of *P. xanthii* by spraying zucchini plants with TAET1. Mean proportion of leaf area with powdery mildew symptoms per plant, percentage of the number of leaves with symptoms per plant, and percentage of number of petioles with powdery mildew. Px: Application of *P. xanthii*. Px-TAET1: application of TAET1 3 days before application of *P. xanthii*. Data were tested using Student’s *t*-test. * significant at *p* < 0.05.

In turn, foliar application of TAET1 considerably suppressed the disease caused by *P. xanthii* (Figure 9), with a reduction of 66.78% in the proportion of leaf area showing symptoms. Similarly, the number of leaves and petioles with powdery mildew was reduced by 31.42% and 33.39%, respectively.

## 4. Discussion

Species of the genus *Trichoderma* are of great interest for their benefits in agriculture and natural ecosystems. *Trichoderma* species have antagonistic activity against several soil-borne and aerial-borne plant pathogens, mainly fungi such as *Fusarium oxysporum* [31,71,72,73,74,75], *F. solani* [76,77,78], *B. cinerea* [79,80,81,82,83,84,85], *S. sclerotiorum* [82,86], *S. minor* [87], *Rhizoctonia solani* [72,88,89,90], *Phytophthora capsici* [91,92], *Phytophthora* *parasitica* [85], *Chondrostereum purpureum* [93], *Macrophomina phaseolina* [94], *Podosphaera xanthii* [95], *Alternaria alternata*, [96], *Pythium aphanidermatum* [97] and *Pythium ultimum* [24,98], among many others.

In the present study, the isolate *T. aggressivum* f. *europaeum* TAET1 was selected to control different phytopathogens of interest in horticultural crops, assessing percentages of mycelial growth inhibition and disease severity reduction higher than those found in other *Trichoderma* species. For example, Zhang et al. [99] reported 75% and 82% in vitro mycelial growth inhibition of *B. cinerea* and *R. solani* by *T. longibrachiatum*, respectively. Fernandes et al. [100] also found good results of mycelial growth inhibition of *S. sclerotiorum*, *R. solani*, and *F. solani* when using different *Trichoderma* species. The same authors reported that *T. tomentosum* showed efficient antagonism against *S. sclerotiorum* and *R. solani* and moderate antagonism against *F. solani.* Additionally, Amin et al. [101] reported that *T. viride* highly inhibited the mycelial growth of *R. solani*, *S. rolfsii,* and *S. sclerotiorum* in comparison with *T. harzianum*. In general, the results from the comparison vary with the *Trichoderma* isolate and with the phytopathogen. Additionally, the results are affected by the growth medium, temperature, and other factors. Similar results were found in in vivo tests and in aerial or soil disease control. Rini and Sulochana [102] achieved 25% *Rhizoctonia* root rot incidence control after applying *T. harzianum* or *T. pseudokoningii*. In turn, Hafez et al. [103] reported approximately 40% control of powdery mildew caused by *P. xanthii* in cucumbers when using T. *harzianum* and *T. viride.* Both disease control percentages are lower than the values reached in this study with *T. aggressivum*. Very few studies have been conducted using *T. aggressivum* f. *europaeum* as the biological control agent. Sánchez-Montesinos et al. [24] have reported results very similar to those found in this study when analysing *P. ultimun* after applying *T. aggressivum* f. *europaeum*, even under saline stress conditions in melon plants. In *F. solani* f. *cucurbitae*, although the disease has not been well controlled, in contrast to the in vitro results, the disease onset was considerably delayed. Khanzada et al. [104] reported an in vitro mycelial growth inhibition close to 20% in comparison to *T. harzianum*. This percentage was higher for other species, reaching values close to 70% for *T. pseudokoningii.* Pérez-Hernández et al. [105] reported that applying *T. harzianum* strain T22 had no effect on disease control, in contrast to Roberti et al. [106], who detected *Fusarium* crown and foot rot control when applying the same strain, T22, and other formulations based on *Trichoderma*, in a range lower than 30%.

Interactions have been detected between *T. aggressivum* f. *europaeum* TAET1 hyphae and different pathogens. Hyperparasitism and strong competition exerted because of rapid growth and sporulation have been the main inhibition mechanisms reported in the literature [25]. Compared to the results from other authors, little antagonistic activity because of volatile metabolites was observed in this study. Recently, the production of volatile organic compounds (VOC) of green mould (*T. aggressivum* f. *europaeum*) on different culture media have been described by Radványi et al. [107]. Similarly, Krupke et al. [108] described the production of the metabolite 3,4-dihydro-8-hydroxy-3-methylisocoumarin by *T. aggressivum*. Lee et al. [59] established the relationship between VOC production by *T. aggressivum f. europaeum* and *Arabidopsis* growth promotion. Pandey et al. [109] reported enzyme production and VOC effects on *Sclerotium rolfsii, R. bataticola, F. oxysporum, F. udum,* and *Colletotrichum capsici* mycelial growth, assessing inhibition percentages ranging from 8 to 55% in vitro. Different studies on *T. harzianum* have demonstrated that the production of volatile metabolites resulted in in vitro mycelial growth inhibition percentages higher than 50% for *F. oxysporum* f. sp. *dianthi* [110]. Similar results were found by El-Katatny et al. [111] because of overproduction of volatile metabolites against *Sclerotium rolfsii.*

Studies on *T. aggressivum* f. *europaeum* sensitivity to fungicides have generally focused on fungicides used to control green mould in cultivated mushrooms, such as prochloraz and metrafenone [112]. Unlike some results from this work, tests conducted by Kosanovic et al. [42] reported that *T. aggressivum* f. *europaeum* isolates were sensitive to chlorothalonil and carbendazim and less susceptible to iprodione, and some isolates were resistant to thiophanate-methyl and resistant to trifloxystrobin. Williams et al. [51] developed a selective medium for *Trichoderma* isolation from commercial *Agaricus bisporus* composts containing propamocarb and discarded captan for inhibiting sporulation. Other studies have analysed the sensitivity to chemical active substances or biological agents [49], and *T. aggressivum* f. *europaeum* has been applied as a biocontrol agent only sporadically; therefore, no compatibility studies have been performed on this species with fungicides commonly used to control diseases in horticultural crops. Our study provides information on the compatibility of *T. aggressivum* f. *europaeum* TAET1 against different doses of fungicides in vitro. However, new studies must be conducted *in planta* to better establish the limits of each and to enable their use in integrated disease management.

There is no consensus on the use of pathogens derived from cultivated mushrooms [113,114]. Numerous commercial biofungicide formulations are based on species that have been described as causal agents of green mould. Similarly, although different *Trichoderma* species have been described as pathogenic agents for some plant species, such as pine seedlings [115], or as a pathogen causing ear rot disease in maize [116], and even as the cause of disease in humans after eating contaminated food [117,118] or in immunosuppressed patients [119,120], the use of *Trichoderma* in agriculture can be considered one of the best alternatives to chemical control because its benefits far outweigh any phytosanitary disadvantage.

## 5. Conclusions

Accordingly, based on the results from our study, we consider that *T. aggressivum* f. *europaeum* TAET1 should be studied in commercial greenhouses for future commercial purposes. Applications as a preventive and/or control of fungal plant diseases may be a viable alternative to the use of conventional synthetic compounds. The high compatibility of this isolate with fungicides could allow its use in combination with different pest management strategies.

## Figures and Tables

**Table 1:** Effect of different fungicides on mycelial growth of *P. variotii* at different doses (D1–D4). Mean values (±standard deviation) followed by different letters (line) indicate significant differences (*p* < 0.05) by least significant difference test (LSD). (1) Cytoskeleton and motor proteins, (2) signal transduction, (3) sterol biosynthesis in membranes, (4) amino acids and protein synthesis, (5) respiration, (6) lipid synthesis or transport/membrane integrity or function, (7) chemicals with multi-site activity, (8) host plant defence induction, (9) unknown mode of action, (10) nucleic acids metabolism, and (11) cell wall biosynthesis. Fungicide/fungistactic action (red letters): F: Fungicide; f: fungistactic.

			Doses (ppm)							
			D1 (0.5 × D2)		D2		D3		D4 (1.5 × D3)	
Fungicide—Action Mechanisms	D2	D3	Growth	Inhib.	Growth	Inhib.	Growth	Inhib.	Growth	Inhib.
Thiophanate-methyl 70% (WP) *w*/*w*—(1) (F: D1 D2 D3 D4)	500	1000	0 ± 0a	100.0%	0 ± 0a	100.0%	0 ± 0a	100.0%	0 ± 0a	100.0%
Pencycuron 25% (SC) *w*/*v*—(1)	5000	8000	55.8 ± 3.3a	19.1%	52 ± 1.6b	24.6%	50.8 ± 1.3b	26.4%	46 ± 0.7c	33.3%
Iprodione 50% (SC) *w*/*v*—(2)	1000	1500	63 ± 2.3a	8.7%	53 ± 1.6b	23.2%	43.6 ± 4.5c	36.8%	33.4 ± 5d	51.6%
Flutriafol 12.5% (SC) *w*/*v*—(3)	2000	2500	23.4 ± 1.7a	64.7%	19.4 ± 1.3b	70.7%	18.3 ± 1.2bc	72.4%	16.6 ± 2.6c	74.9%
Triadimenol 25% (EC) *w*/*v*—(3)	250	500	61.8 ± 3.7a	6.6%	55 ± 4.5b	16.9%	39 ± 3.1c	41.1%	34.2 ± 1.3d	48.3%
Myclobutanil 24% (EC) *w*/*v*—(3)	200	400	23.8 ± 0.8a	52.2%	16 ± 1.4b	67.9%	5.6 ± 0.5c	88.8%	3.4 ± 0.5d	93.2%
Fenhexamid 50% (WG) *w*/*w*—(3)	1500	2000	30 ± 0.7a	53.6%	26.8 ± 0.8b	58.5%	25.2 ± 0.4c	61.0%	23.4 ± 0.5d	63.8%
Pyrimethanil 40% (SC) P/V—(4)	1500	2000	5 ± 0a	92.7%	3.62 ± 0.4b	94.7%	2.28 ± 0.4c	96.7%	1.5 ± 0.9c	97.8%
Azoxystrobin 25% (SC) *w*/*v*—(5)	800	1000	35.3 ± 2.3a	46.7%	33.3 ± 1b	49.7%	31.2 ± 0.8bc	52.9%	30.7 ± 1.2c	53.6%
Kresoxim-methyl 50% (WG) *w*/*w*—(5)	200	500	60.4 ± 6.1a	14.0%	58.8 ± 7.7a	16.2%	49.3 ± 1.2b	29.8%	45.9 ± 0.7b	34.6%
Propamocarb 60.5% (SL) *w*/*v*—(6)	2500	5000	66.4 ± 1.5a	3.8%	54.6 ± 9.3b	20.9%	29.8 ± 4.1c	56.8%	21 ± 2.9d	69.6%
Copper hydroxide 35% (WG) *w*/*w*—(7)	2000	3000	24 ± 1.2a	62.8%	16.8 ± 1.5b	74.0%	15.2 ± 1.3b	76.5%	9.4 ± 0.5c	85.4%
Mancozeb 80% (WG) *w/w*—(7) (f: D2D3) (F: D4)	2000	3000	7 ± 7.3a	85.9%	0 ± 0b	100.0%	0 ± 0b	100.0%	0 ± 0b	100.0%
Chlorothalonil 50% (SC) *w*/*v*—(7)	2500	3000	3.6 ± 1.5a	94.6%	3 ± 1.2ab	95.5%	1.8 ± 0.8bc	97.3%	1 ± 1.2c	98.5%
Fosetyl-AL 80% (WG) *w*/*w*—(8)	2500	3000	45.2 ± 1.8a	9.2%	33 ± 0.7b	33.7%	27.8 ± 1.8c	44.2%	19.2 ± 1.3d	61.4%
Cymoxanil 60% (WG) *w*/*w*—(9)	200	300	70 ± 1a	−8.4%	65.8 ± 1.3b	−1.9%	61.2 ± 2.8c	5.3%	57.8 ± 2.4d	10.5%
Cyprodinil 37.5% + Fludioxonil 25% (WG) *w*/*w*—(4 + 2)	600	1000	7.8 ± 1.8a	84.3%	6.4 ± 0.5a	87.1%	4.6 ± 0.5b	90.8%	4.7 ± 1b	90.6%
Folpet 40% + Metalaxyl-M 10% (WP) *w*/*w—*(7 + 10)	2000	2500	14.6 ± 0.5a	78.8%	12.3 ± 0.7b	82.2%	10.2 ± 0.8c	85.2%	7.4 ± 0.5d	89.3%
Dimethomorph 7.5% + Mancozeb 66.7% (WG) *w*/*w*—(11 + 7)	2000	3000	53.6 ± 1.5a	22.3%	49.8 ± 1.3b	27.8%	45.8 ± 0.8c	33.6%	43.4 ± 1.1d	37.1%

**Table 2:** Antagonistic potential of *T. aggressivum* f. *europaeum* isolates against six phytopathogens in dual culture on PDA medium. % mycelial inhibition was calculated as PIRM = (R1 − R2) ÷ R1 × 100, where: PIRM: percentage inhibition of radial mycelia growth of the pathogen, R1: radial growth of pathogen in control plates, R2: radial growth of pathogen in dual culture plates. Means with the same letter are not significantly different (LSD) according to ANOVA test (*p* < 0.05).

	% Inhibition of Mycelial Growth Plant Pathogens					
Isolates	*Botrytis cinerea*	*Sclerotinia sclerotiorum*	*Fusarium solani*	*Rhizoctonia solani*	*Mycosphaerella melonis*	*Pythium aphanidermatum*
**TAE492**	74.25 ± 1.11 ^d^	85.25 ± 0.55 ^a^	84.25 ± 1.42 ^e^	80.75 ± 2.27 ^c^	87.25 ± 2.05 ^ab^	63.5 ± 0.55 ^b^
**TAE493**	76.5 ± 2.40 ^bcd^	84.00 ± 0.55 ^ab^	85.75 ± 0.68^cd^	81.75 ± 1.42 ^bc^	87.25 ± 2.03 ^cd^	63.75 ± 0.88 ^ab^
**TAE880**	76.75 ± 1.11 ^abcd^	84.25 ± 1.42 ^ab^	86.00 ± 0.55 ^bcd^	80.50 ± 0.68 ^c^	87.00 ± 1.11 ^de^	55.00 ± 1.25 ^e^
**TAE1320**	70.25 ± 2.4 ^e^	83.75 ± 1.25 ^abc^	82.00 ± 0.68 ^f^	76.00 ± 1.04 ^e^	85.50 ± 1.42 ^b^	58.5 ± 1.04 ^c^
**TAE1409**	77.5 ± 3.30 ^abcd^	83.5 ± 1.36 ^abcd^	85.75 ± 1.42 ^cd^	81.50 ± 1.04 ^bc^	87.00 ± 1.11 ^ab^	65.00 ± 0.88 ^a^
**TAEB80**	78.25 ± 3.81 ^abc^	81.75 ± 3.37 ^bcd^	86.50 ± 0.55 ^bcd^	80.50 ± 0.68 ^c^	86.75 ± 2.59 ^ab^	53.5 ± 0.55 ^f^
**TAET1**	80.00 ± 1.76 ^a^	84.00 ± 1.62 ^ab^	89.75 ± 2.85 ^a^	82.75 ± 0.55 ^ab^	87.50 ± 0.88 ^ab^	63.00 ± 1.11 ^b^
**TAE13**	75.00 ± 5.79 ^cd^	83.00 ± 2.27 ^abcd^	87 ± 0.68 ^bc^	76.75 ± 1.42 ^e^	88.25 ± 2.09 ^a^	59 ± 1.04 ^c^
**TAET2**	75.50 ± 1.11 ^cd^	83.25 ± 3.13 ^abcd^	85.50 ± 0.68 ^de^	78.50 ± 1.36 ^d^	87.25 ± 2.05 ^ab^	54.25 ± 1.42 ^ef^
**TAET3**	76.75 ± 1.42 ^abcd^	81.00 ± 2.40 ^cd^	87.25 ± 0.55 ^b^	82.25 ± 0.55 ^ab^	87.00 ± 0.68 ^ab^	58 ± 2.27 ^cd^
**TAET4**	79.00 ± 1.04 ^ab^	82.25 ± 4.27 ^bcd^	89.25 ± 0.68 ^a^	82.25 ± 0.55 ^ab^	88.75 ± 0.88 ^a^	65.00 ± 0.88 ^a^
**TAET5**	78.00 ± 2.27 ^abc^	80.75 ± 1.89 ^d^	86.5 ± 0.55 ^bcd^	83.25 ± 0.68 ^a^	86.75 ± 0.68 ^ab^	56.75 ± 0.68 ^d^
***P***	*0.0001*	*0.0818*	*0.0000*	*0.0000*	*0.2860*	*0.0000*

Mean values (±standard deviation) followed by different letters (line) indicate significant differences (*p* < 0.05) by least significant difference test (LSD).

**Table 3:** Mycelial growth (cm) of phytopathogens on PDA medium due to exposure to *Trichoderma aggressivum* f. *europaeum* TAET1 volatiles vs. the growth control. * Means are significantly different (LSD) according to *t* students test (*p* < 0.05).

	Mycelial Growth (cm) of Plant Pathogens					
Isolates	*B. cinerea*	*S. sclerotiorum*	*F. solani*	*R. solani*	*M. melonis*	*P. aphanidermatum*
**Control**	4.26 ± 0.05	4.36 ± 0.11	4.26 ± 0.05	4.25 ± 0.05	4.20 ± 0.17	4.40 ± 0.10
**TAET1**	4.20 ± 0.17	4.16 ± 0.23	3.50 ± 0.40 *	4.10 ± 0.23	3.66 ± 0.25 *	4.23 ± 0.11
***p***	*0.5614*	*0.2508*	*0.0303*	*0.2377*	*0.0390*	*0.2901*

**Table 4:** Effects of applying *T. aggressivum* f. *europaeum* TAET1 on sclerotia of *S. sclerotiorum.* Number of sclerotia recovered, percentage of sclerotial germination, and TAET1 colonisation measured after 25 d of incubation. * Values represent means of five replicates. Data were analysed using Student’s *t*-test (*p* < 0.05).

	Sclerotia Recovered	Sclerotial Germination (%)	TAET1 Colonization (%)
Control	5	63.2	0
TAET1	2.1 *	0 *	36.8 *

## Data Availability

Not applicable.
